# Grazing Allometry: Anatomy, Movement, and Foraging Behavior of Three Cattle Breeds of Different Productivity

**DOI:** 10.3389/fvets.2020.00494

**Published:** 2020-08-14

**Authors:** Caren M. Pauler, Johannes Isselstein, Joel Berard, Thomas Braunbeck, Manuel K. Schneider

**Affiliations:** ^1^Forage Production and Grassland Systems, Agroscope, Zurich, Switzerland; ^2^Department of Crop Sciences, Georg-August-University, Göttingen, Germany; ^3^Centre for Organismal Studies, Ruprecht-Karls-University, Heidelberg, Germany; ^4^AgroVet-Strickhof, Lindau, Switzerland; ^5^Animal Production Systems and Animal Health, Agroscope, Zurich, Switzerland

**Keywords:** alpine pastures, cattle breeds, claws, forage selection, GPS, movement behavior, pedometer, species diversity

## Abstract

Modern breeding has formed a multitude of cattle breeds ranging from undemanding, low-productive breeds to high-productive, specialized dairy, or beef cattle. The choice of breed has important implications for farm management, but its impact on pasture vegetation is underestimated. We hypothesized (i) that anatomy, movement, and foraging behavior of cattle are allometrically related on the individual level, (ii) that differences among cattle are not explained by individual variation alone but also by breed, and (iii) that anatomy, movement, and foraging behavior of a cattle breed is related to its productivity. In order to test these hypotheses, we conducted a controlled grazing experiment in which three cattle breeds simultaneously grazed three types of heterogenous, alpine pastures: low-productive Highland cattle (average weight: 358 kg); local, dual-purpose Original Braunvieh (582 kg); and high-productive Angus × Holstein crossbreed (679 kg). We measured body weight and claw base of nine cows per breed after 10 weeks of grazing alpine pastures. Over a period of 9 days, we recorded the step frequency and lying time by pedometer and space use by GPS. Moreover, we visually observed foraging behavior on three occasions per cow. Forage selectivity and quality were calculated for every cow's diet. Allometric relationships were analyzed on the individual level by fitting standardized major axes. For most parameters measured, we detected strong allometric relationships and clear differences among breeds that depended on the level of productivity. The claws of Highland cattle were relatively large compared to their body weight and thus they exerted less static pressure than other breeds. Moreover, the more productive a breed was, the higher its selectivity and step frequency were. For example, Highland cattle foraged shrubs and thistles more frequently than high-productive Angus × Holstein. The latter walked longer distances to select higher-quality forage, while Highland cattle used the space more evenly, visited steeper slopes, and moved further away from water points. Irrespective of breed, vegetation composition influenced cattle behavior: On pastures of low forage quality, animals walked more, foraged more selectively, and used space less evenly. In conclusion, the observed breed-specific differences can be used to improve pasture management and grassland conservation.

## Introduction

The domestication of wild aurochses (*Bos primigenius*) created a plethora of cattle breeds (*Bos taurus*) with different characteristics (1). While the aurochs slowly evolved to cope with environmental conditions (2), human breeding decisions enormously accelerated genetic transformation to meet agricultural needs, and adaptation to the natural environment became less important (3). During the mid-nineteenth century, different breeds emerged from pure-breeding, as motivated by ideas of Darwinism, Mendelism, and biometry. In recent decades, genetic improvements facilitated by artificial insemination, quantitative genetics, and molecular markers considerably increased productivity (4). Thereby, traits prioritized by humans, particularly milk yield, body weight, feed intake, and growth rate were enhanced. Records of historical livestock production in Austria indicate that at the beginning of nineteenth century cows weighed about 250 kg and produced 1,300 kg of milk per year (5). Today, specialized beef cattle, such as Charolais or Blonde d'Aquitaine, weigh about 700–950 kg (6), and specialized dairy cows, such as Holstein Friesian, produce up to 12,800 kg of milk per year when fed concentrates (7). In addition to these prioritized traits, which breeding controls, there are numerous characteristics that are not accounted for in selection and have co-evolved unnoticed. Some of these hidden traits recently have gained awareness, such as robustness (8), longevity, and feed efficiency (9), while others, such as claw size, movement, and foraging behavior, have long been ignored in herdbook breeding (10).

Such profound transformations of cattle are likely to have an impact on the vegetation of the sites they graze. Semi-natural pastures, which belong to the most diverse habitats on earth (11), were created by centuries of low-intensity grazing with low-productive animals (12). If the animals that formed these pastures undergo tremendous modifications within a few decades, vegetation may also change. Indeed, in a recent study we identified differences in vegetation when pastures were grazed by breeds of different productivity (13). In order to quantify the drivers of these differences, a follow-up study was designed: Strong changes in body weight, e.g., may exert increased pressure to the ground with negative consequences for vegetation, soil properties, and claw health. Cattle's claws are particularly interesting, because the base that is burdened by animal mass was not considered in breeding decisions and is, therefore, presumably disproportionately underdeveloped. Moreover, higher body weight, growth rate, and milk yield probably altered movement and foraging behavior. If modern cattle walk more, use the pasture differently, or forage other plants than their lower-productive ancestors, this could influence vegetation composition, as suggested by Pauler et al. (14).

Unfortunately, it is not possible to compare modern, high-productive cattle directly to their low-productive ancestors, which grazed pastures centuries ago, before production-oriented herdbook breeding began. However, there are modern, low-productive breeds, such as Highland cattle, which are less affected by breeding: Mason (3) postulated little difference between modern Highland cattle and sculptures of cattle made by ancient Etruscans. While other breeds annually broke records of beef and milk production, the main breeding aim of Highland cattle was to thrive under harsh environmental conditions and on the low forage quality of the Scottish Highlands. Consequently, these animals are lighter and grow more slowly; at the same time, however, they are more robust and less demanding than high-productive breeds (15).

If productivity of cattle has an impact on pasture vegetation, there are far-reaching consequences for habitat conservation of low-productive grasslands, which host many vulnerable and endangered plant species (11, 16). These species may be negatively affected by grazing with high-productive cattle breeds as suggested by Pauler et al. (13): Plant species resistant to selective foraging, such as thistles or shrubs, as well as species adapted to trampling become dominant on pastures of high-productive breeds, and thus biodiversity decreases (13, 17). Moreover, in contrast to Highland cattle, high-productive animals are insufficiently alimented by the forage present in low-productive grasslands (18).

In the present study, we hypothesized (i) that anatomy, movement and foraging behavior of cattle are allometrically related on the individual level, (ii) that differences among cattle are not explained by individual variation alone but also by breed, and (iii) that anatomy, movement and foraging behavior of a cattle breed are related to its productivity. These hypotheses were tested, for the first time, in a controlled grazing experiment on species-rich alpine pastures using three cattle breeds that differ widely in productivity.

## Materials and Methods

### Three Breeds: Low-, Medium-, and High-Productive

We investigated anatomy, movement, and foraging behavior of three cattle breeds, representing a gradient from low to high productivity: The lower end of this gradient was represented by Highland cattle (HC), an undemanding and low-productive traditional breed, bred to thrive in the harsh environmental conditions of the Scottish Highlands, but widespread all over the world. Cattle of medium productivity were represented by Original Braunvieh (OB), a dual-purpose breed traditionally kept in the Swiss Alps, with body weight, and growth rate considerably higher than that of Highland cattle (6). The Original Braunvieh is not to be confused with Brown Swiss, a high-productive, but genetically less diverse dairy breed selected from the same original population (19). The most productive breed in our experiment was Angus × Holstein crossbreed (AH), which combines the strongly muscled, heavy body of Angus beef cattle with the large-framed body and elevated milk production of Holstein dairy cows.

The cows taking part in the experiment were randomly selected from their original herds. All cows were familiar with mountainous grasslands, since they originated from mountain farms and also, had experience grazing high-elevation, alpine pastures in preceding summers. At their home farms, all study animals were fed grass silage and hay only. They were kept in the stable during winter with regular access to pastures in spring. Due to similar previous forage experience and housing conditions, we assumed that breeds experienced similar pre-conditioning. Cows were aged between 2.8 and 10.3 years (HC: 80 months, range: 53–124; OB: 46 months, range: 34–75; AH: 92 months, range: 60–110). We tested all variables for correlations with age, but found only weak relationships (*R*^2^: 0.08–0.31).

Over a period of 2 weeks before the experiment was started, all cattle were allowed to graze the pastures of the study area together to acclimatize to the alpine conditions. For each of the breeds studied, three subgroups of three suckler cows and their calves were formed, resulting in a total of 54 animals. The subgroups were developed by ranking cows breed-wise based on specific body weight and joining every third individual (1 heavy, 1 middle weight, 1 light cow per subgroup). Anatomy and behavior were quantified for the 27 cows, but not for the calves.

### Study Areas: Three Types of Alpine Pastures

**Figure 1:**
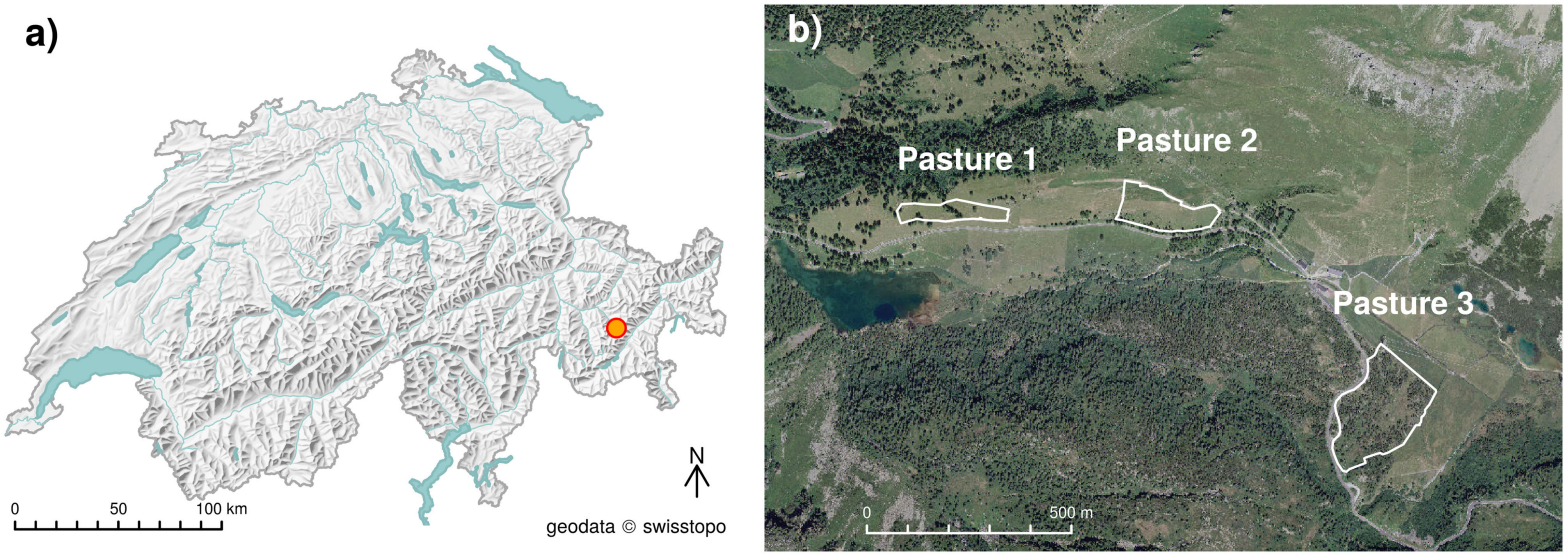
**(a)** Overview map and **(b)** aerial image of the study area in Swiss Alps with the three pastures grazed by cattle in the experiment.

Movement and forage behavior were observed on three types of alpine pastures on Alp Weissenstein in the eastern Swiss Alps (2,026 m asl., 46.5816°N, 9.8002°E, Figure 1).

The three pastures differed in plant species composition, forage quality, and bedrock material (Tables 1, 2). We calculated a total forage demand of 1,800 kg dry matter per pasture for all individuals during the experiment. In spring, the pastures already supplied 2,440–4,860 kg dry matter and there was additional regrowth during summer. Pasture size was set to provide excessive forage compared to the estimated forage demand and the actual biomass was measured by a rising plate meter (14). This amount of excess forage made sure that cattle selected plants based on preference rather than being pressured by shortage.

**Table 1:** Characterization of the three pastures the cattle were grazed on.

	**Pasture 1**	**Pasture 2**	**Pasture 3**
Description	Nutrient-rich, flat	Heterogeneous, steep with few flat parts, nutrient-poor with few nutrient-rich parts	Steep wood pasture, flat fens, extremely nutrient-poor
Bedrock material	Calcareous	Calcareous	Crystalline
Slope (%)^a^	19.2; 0.3; 56.7	48.1; 1.2; 122.6	25.1; 0.3; 146.7
Size (ha)	1.05 (0.39; 0.39; 0.27)	1.83 (0.70; 0.69; 0.43)	4.38 (1.66; 1.71; 1.01)
Stocking density (LU/ha)	11.4 (12.3; 12.2; 9.8)	6.6 (6.8; 7.0; 6.1)	2.8 (2.9; 2.8; 2.7)
Stocking rate (LU/ha/a)	0.28	0.20	0.08
Forage quality	High (5.9)^b^	Medium (4.6)^b^	Low (2.7)^b^
Available biomass (kg DM)	3,380	2,440	4,860^c^
Vegetation type (Table 2)	• Fertile pasture	• Fertile pasture • Mat-grass community • Dwarf-shrub community	• Alpine fen • Larch-Pine forest

*Table provides a short description of each pasture; the predominant bedrock; the average, minimum, and maximum slope (%)^a^; the total size (ha) of the entire pastures and, in brackets, the average paddock size of Angus × Holstein, Original Braunvieh, and Highland cattle; the stocking density (LU/ha) normalized to the metabolic live weight (= weight^0.75^) of a cow of 600 kg, on average and, in brackets, for the three breeds; the total stocking rate (LU/ha/yr); the forage quality relative to the other pastures and as averaged forage indicator value (21); the available biomass (kg dry matter); and the main vegetation type (20)*.

a *SwissAlti3D, Federal Office of Topography swisstopo, Wabern*.

b *Average cover-weighted mean of forage quality indicator value (21) of all vascular plant species within 18 vegetation subplots per pasture, estimated before the first grazing in spring. For details, see Pauler et al. (14)*.

c *Total standing biomass including woody structures in the herb layer (mainly dwarf shrubs)*.

**Table 2:** Characterization of the vegetation types.

**Vegetation type**	**Association**	**Dominant plant species**
Fertile pasture	*Poion alpinae*	*Trifolium pratense* L. *Trisetum flavescens* (L.) P. BEAUV. *Phleum rhaeticum* (HUMPHRIES) RAUSCHERT *Ranunculus acris* L. *Carum carvi* L. *Alchemilla xanthochlora* ROTHM.
Mat-grass community	*Nardion*	*Festuca rubra* L. *Nardus stricta* L.
Dwarf-shrub-community	*Juniperion nanae*	*Erica carnea* L. *Calluna vulgaris* (L.) HULL
Alpine fen	*Caricion fuscae*	Various mosses *Trichophorum cespitosum* (L.) HARTM. *Carex nigra* (L.) REICHARD *Carex panicea* L.
Larch-pine forest	*Larici-Pinetum cembrae*	*Larix decidua* MILL. *Pinus cembra* L. *Vaccinium myrtillus* L. *Vaccinium gaultherioides* BIGELOW *Juniperus communis* L.

*Table provides the main vegetation types in the study area [classification according to Delarze and Gonseth (20)], the scientific name of these plant associations, and their dominant plant species*.

The three pastures were (i) a nutrient-rich, flat pasture, (ii) a steep, nutrient-poor pasture with few flat and nutrient-rich areas, and (iii) an extremely nutrient-poor, steep wood pasture with flat fens (for details, see Tables 1, 2). Each pasture was subdivided into three paddocks with highly comparable conditions (14). The three paddocks of a pasture were grazed simultaneously by three subgroups—one of each breed. The paddock size was adjusted to the breed to ensure similar stocking density despite the lower body weight and forage demand of Highland cattle (Table 1). Thus, additional space was added to the paddocks of Original Braunvieh and Angus × Holstein. Stocking density and stocking rate were calculated by normalizing metabolic body mass (= weight^0.75^) to cows of 600 kg (22).

From the three paddocks of pasture 1, the three subgroups (= three cows plus calves per breed) were transferred to the paddocks in the second, and subsequently in the third pasture. The animals stayed 3–4 days on each pasture. This rotation procedure was repeated three times. Different subgroups and, therefore, different animals were used for each rotation to avoid pseudoreplication. Applying a Latin square design, a different breed grazed each paddock in each rotation. Thereby, each breed visited each paddock once. This procedure resulted in three independent repetitions to account for variation in social behavior, season, and weather. On each pasture, movement and foraging behavior of every cow in the subgroup were observed. During the rotations, the remaining animals of the other two subgroups per breed were kept on another pasture not included in the experiment.

The paddocks of pasture 1 were relatively small compared to other alpine farms (23). This was necessary to define homogeneous paddocks. Larger paddocks would have led to confounding effects due to larger heterogeneity. However, the system was not an intensive grazing system since the number of animals per paddock was small. Moreover, the fast rotation reduced stocking rate while allowing for independent replications with different individuals. In Switzerland, the 465, 000 ha alpine pastures are grazed by 300,000 livestock units (LU) for 100 days (23). This results in an average stocking rate of about 0.18 LU/ha/yr. Hence, with 0.08–0.28 LU/ha/yr, the stocking rate in our study was representative of alpine grazing systems in Switzerland (23, 24) and is applicable for extensive grazing systems.

### Assessment of Anatomy: Body Weight and Claw Base

All cows were weighed at the beginning and at the end of the grazing experiment (Weighing System FX15, Texas Trading, Windach, Germany). The body weight after 10 weeks of grazing alpine pastures was used for analysis. The average change in body weight during the grazing period was calculated for each cow.

**Figure 2:**
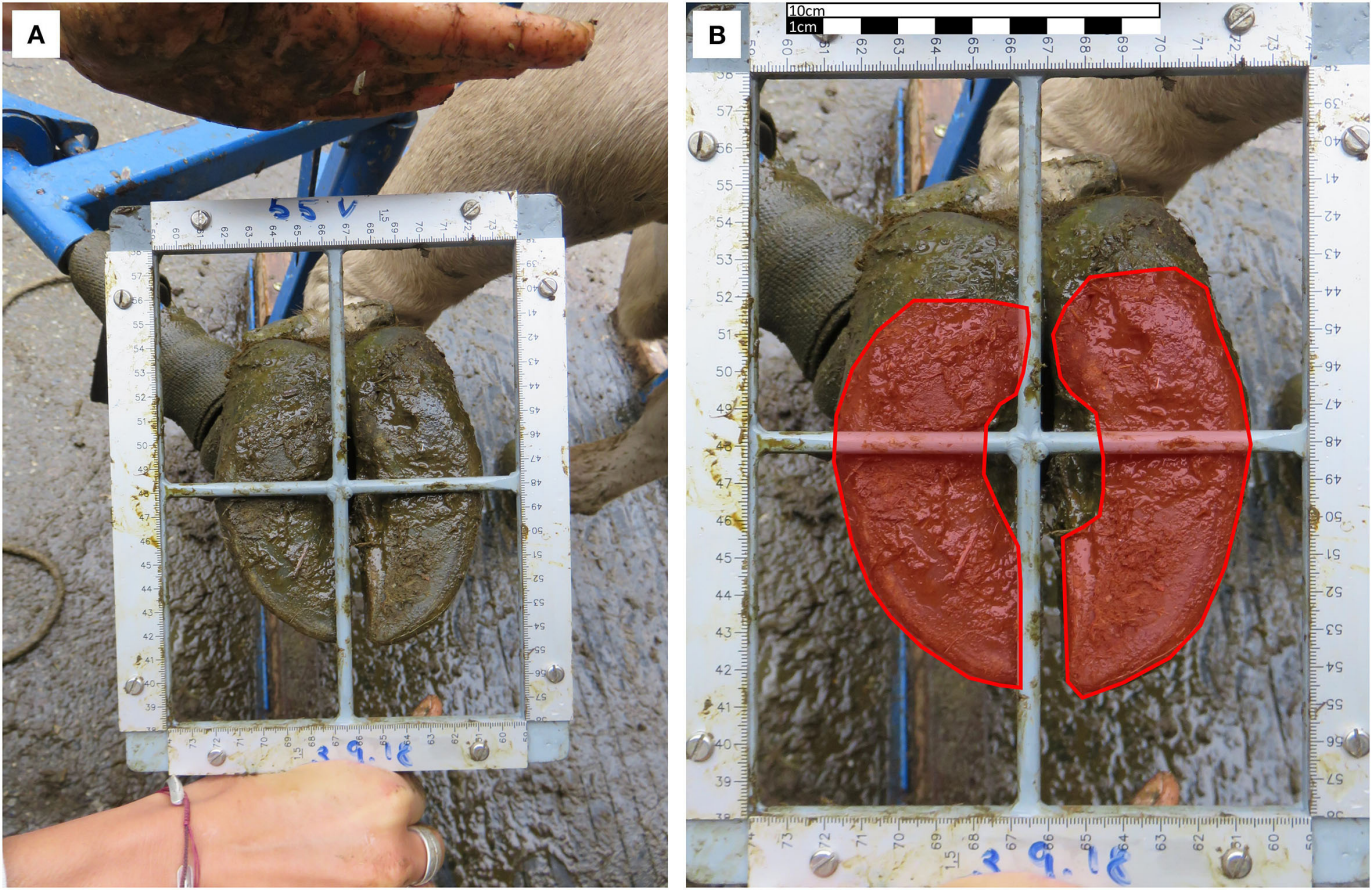
Measurement of claw base: **(A)** Unedited photograph of the ground of a cow's left forefoot with scaled frame. **(B)** The same photograph after rectifying with scale and red polygons, drawn to measure the base of the lateral (on left here) and the medial (on right here) claw.

Two weeks prior to the experiment, the shape and health status of the claws of all 27 cows were inspected by an approved expert and claws were corrected if necessary. At the end of the grazing season, after 10 weeks under similar conditions, the claw base of each cow was measured using the left forefoot and the left hindfoot. Adapting the method of Nuss and Paulus (25) to living animals, we took a picture of the claw base in a scaled frame (Figure 2A) and rectified the photograph (software: Office Lens, Microsoft, Redmond, USA). Using the software “Measure pictures” (CAD-KAS Kassler Computer Software, Markranstädt, Germany), we traced the outline of the claw base and calculated the area of this polygon based on the scale included in the picture (Figure 2B). Thus, we measured the medial and lateral claws of both feet. Assuming the left claws as proxies for the right claws (26), we doubled the values and summed them. Static pressure to the ground was calculated by dividing the body weight by the summed claw base.

### Assessment of Movement Behavior: Pedometer and GPS Logger

Movement behavior of cows was tracked by pedometers and GPS loggers, which recorded data for the entire duration cows were on the study pastures (9–10 days per cow). To quantify movement behavior, we used IceTag pedometers (IceRobotics, Edinburgh, UK). This device is a three-axis accelerometer that uses the force of movement to identify the number of times a cow lifts its leg and records these events as steps. The time the sensor is horizontal is recorded as lying time. A pedometer was fixed at the left hindfoot of six cows per breed for a total of 18 pedometers installed. The step counts and lying time were recorded for each cow in each pasture separately; from these data, average steps per hour and the proportion of time spent lying (lying ratio) were calculated.

In addition, all 27 cows were equipped with collars carrying a box with a GPS logger (Qstarz BTQ1000XT, Qstarz, Taipei, Taiwan) and 3.6 V lithium batteries (27). The GPS loggers recorded movement for the entire duration cows spent on the study pastures; for unknown reasons, however, 3 of the 27 GPS loggers inadvertently stopped recording prematurely. Positions were logged every 15 s, providing information about the distance covered during a certain time span. The median absolute position error of the GPS devices is 3.1 m (27). The accuracy of covered distance measured by GPS loggers was supported by visual observations and checked against the step count of pedometers, which is not GPS-dependent. The values recorded by pedometers and GPS loggers proved highly correlated (*R*^2^ = 0.90).

For each cow in each pasture, the average hourly covered distance (i.e., speed in m/h) was computed. Furthermore, in order to find out how often cattle visited different portions of the entire available area of each pasture, we calculated the evenness of space use by counting the number of GPS positions within 5 × 5 m grid cells throughout the entire study time and by calculating Camargo's index of evenness across all cells (28). For all cells, two topographic covariates were calculated: percentage slope based on the swissALTI3D digital elevation model with 2 m resolution (Federal Office of Topography, Wabern) and the Euclidean distance to the water sources accessible within the paddock. The positions counted for each individual in each paddock were regressed against each covariate separately using a linear model with a spatially structured and a random error term, fitted using integrated nested Laplace approximation with prior specifications similar to Homburger et al. (23). Covariates were standardized into z-scores to make estimated coefficients comparable across paddocks and individuals.

### Assessment of Foraging Behavior

The foraging behavior was assessed by direct visual observation of the plant species consumed by each cow on 3 different days—one at each pasture type. On each day, every cow was observed foraging for 15–41 min (mean: 26 min), depending on the foraging activity during observation. Before the experiment started, animals were familiarized with to the observer: after a few hours, there was no indication of unnatural behavior and the cows foraged as if they were unobserved. Hence, it was possible to follow the grazing cow in close proximity to the side of the cow's head (from 0.5 to 2 m away). For every second bite, the plant species with the highest share within a bite was recorded. Despite the short distance, it was not always possible to discriminate between some species with similar habitus in the short time available. We therefore combined a few plant species into groups: broad-leaved Poaceae (except *Deschampsia cespitosa*, which was easy to identify, and has much lower forage quality than other broad-leaved Poaceae); fine-leaved Poaceae (except *Nardus stricta*, for which the same applies as for *D. caespitosa*); yellow Asteraceae; *Carex* species; *Trifolium pratense* and *T. repens*; *Potentilla aurea* and *P. erecta*. All other plants were recorded at species level.

Subsequently, we calculated the relative consumption of each plant species or species group per cow and pasture. As a proxy for palatability to cattle, we used the indicator values of forage quality by Briemle et al. (21). The indicator values were multiplied by the relative consumption of all species to estimate the average quality of the consumed forage. For species groups, the relative abundance of the individual plant species within each group in each pasture was calculated based on 186 vegetation relevées (14). In order to reveal how strictly cattle select their forage, we also calculated Pielou's evenness of the selected plant species.

### Statistical Analysis: Tukey Range Tests and Allometric Line Fitting

All calculations were conducted in R 3.6.1 (29). Differences among breeds and among pastures were tested using Tukey range tests as implemented in package *multcomp* (30). For movement variables and foraging behavior of each animal in each pasture, tests were conducted on the mean value per animal over all three pastures, as well as on separate mean values for each pasture. In the text, pairwise comparisons between breeds were labeled by the symbol ~. The effects of paddock size, breed, and their interaction on movement behavior were analyzed by a linear regression model, followed by an analysis of variance.

**Figure 3:**
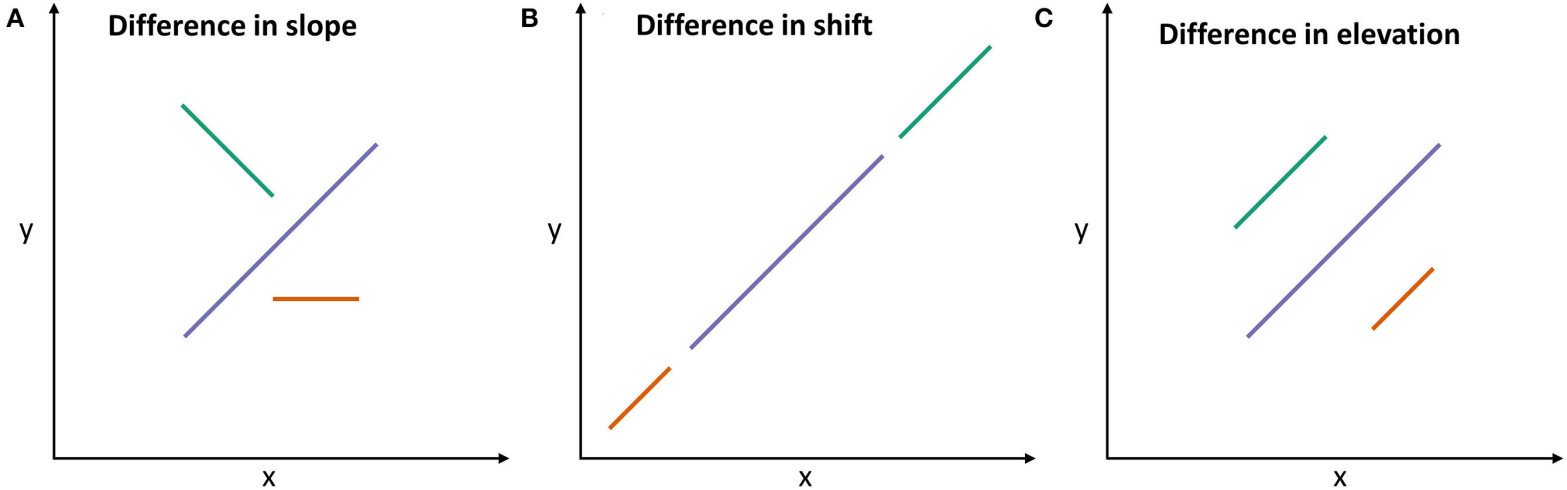
Schematic illustration of differences among allometric lines of three exemplified breeds [adapted from Warton et al. (32)]: **(A)** Allometric lines differ in slope, i.e., the relationships of x and y differ among breeds. **(B)** Allometric lines are shifted along their common slope, i.e., the x and y vary consistently across breeds. **(C)** If allometric lines differ in elevation, they are shifted in parallel to each other, i.e., the values of x differ among groups at similar values of y. The length of allometric lines reflects the data range, but does not affect the allometry.

Allometric relationships on the individual level were estimated by fitting standardized major axes (SMA) using the R package *smatr* (31). SMA is appropriate if there is no causal relationship between two variables x and y, and if x and y differ in variance (32). In contrast to linear regression, SMA minimizes residuals for both axes, not only the y-axis, i.e., both variables are assumed to produce errors. The allometric lines fitted for the three breeds were tested for differences in slope, shift, and elevation. In the case of differing slopes (Figure 3A), the relationship between x and y varied among the three breeds. In cases of a difference in shift (Figure 3B), breeds differed consistently in the levels of x and y. In such cases, breeds had similar values of x at similar values of y. If allometric lines differed in elevation (Figure 3C), the level of the relationship of x and y differed consistently among breeds. In the latter case, breeds had different values of x at similar values of y. For example, in order for the green breed to have a similar elevation as the blue breed, it would have needed to have either larger x or smaller y values.

## Results

### Differences in Body Weight and Claw Base Among Breeds

**Figure 4:**
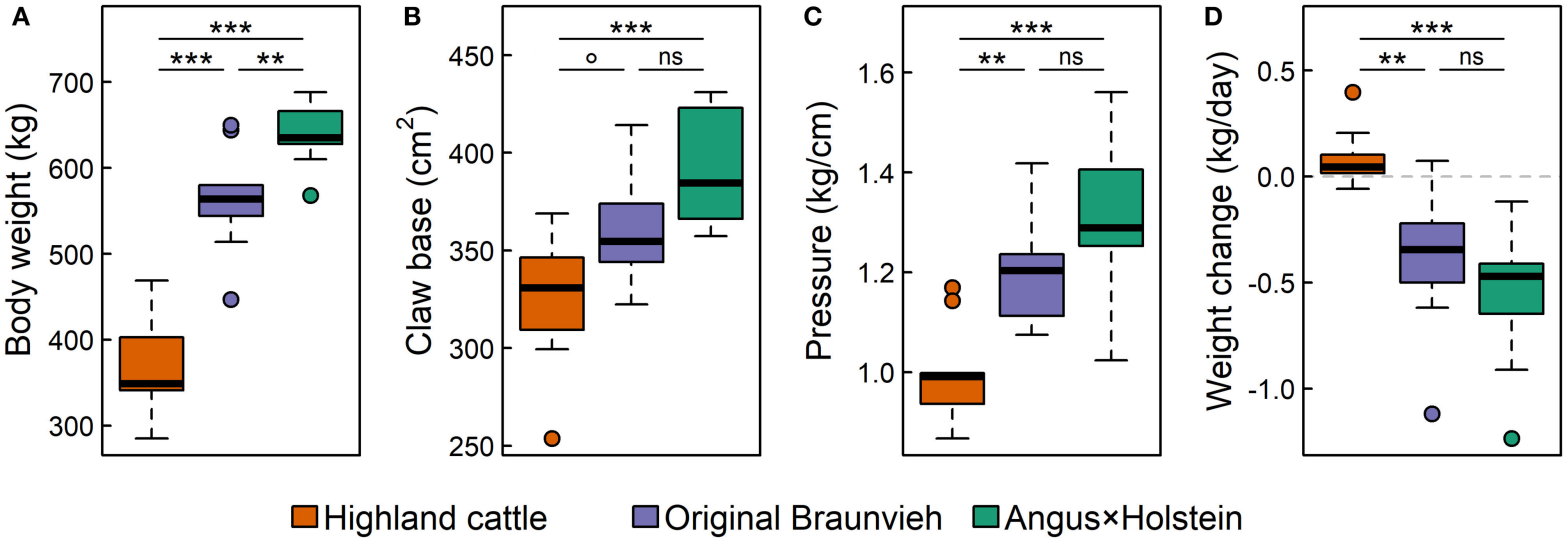
Differences in **(A)** body weight, **(B)** claw base, **(C)** the static pressure of body mass on the ground, and **(D)** the average daily body weight change during 10 weeks on alpine pastures of three cattle breeds: Highland cattle, Original Braunvieh, and Angus × Holstein. Nine cows were measured per breed [box: 25th to 75th quartile range (IRQ); line: median; whiskers: max. 1.5 × IQR; points: outliers; ^ns^*p* > 0.1; °*p* < 0.1; **p < 0.01; ***p < 0.001].

The breeds differed significantly in body weight and claw size (Figures 4A,B). Highland cattle were the lightest breed on the smallest claw base, followed by Original Braunvieh. Angus × Holstein cattle were the heaviest breed and had the largest claws. However, the differences in the claw base were less distinct than the differences in body weight. Hence, claw base generally scaled with body weight, but there were significant differences among breeds beyond individual effects: Although Highland cattle had smaller claws compared to the other two breeds, their claw base was larger relative to their body weight (Figure 4C). Therefore, the static pressure of the body mass on each square centimeter of claw base was significantly lower in Highland cattle than in the other two breeds.

The cattle spent a total of 10 weeks on the alpine pastures, which were relatively nutrient-poor compared to the pastures of their home farms. During this period, Angus × Holstein and Original Braunvieh cattle lost, on average, 0.6 and 0.3 kg weight per day, respectively (Figure 4D). With an average positive daily weight gain of 0.08 kg, Highland cattle differed significantly from the other two breeds (*p*_HC~*OB*_ = 0.002 and *p*_HC~*AH*_ < 0.001, respectively).

### Differences in Movement Behavior Among Breeds as Influenced by Pasture Conditions

**Figure 5:**
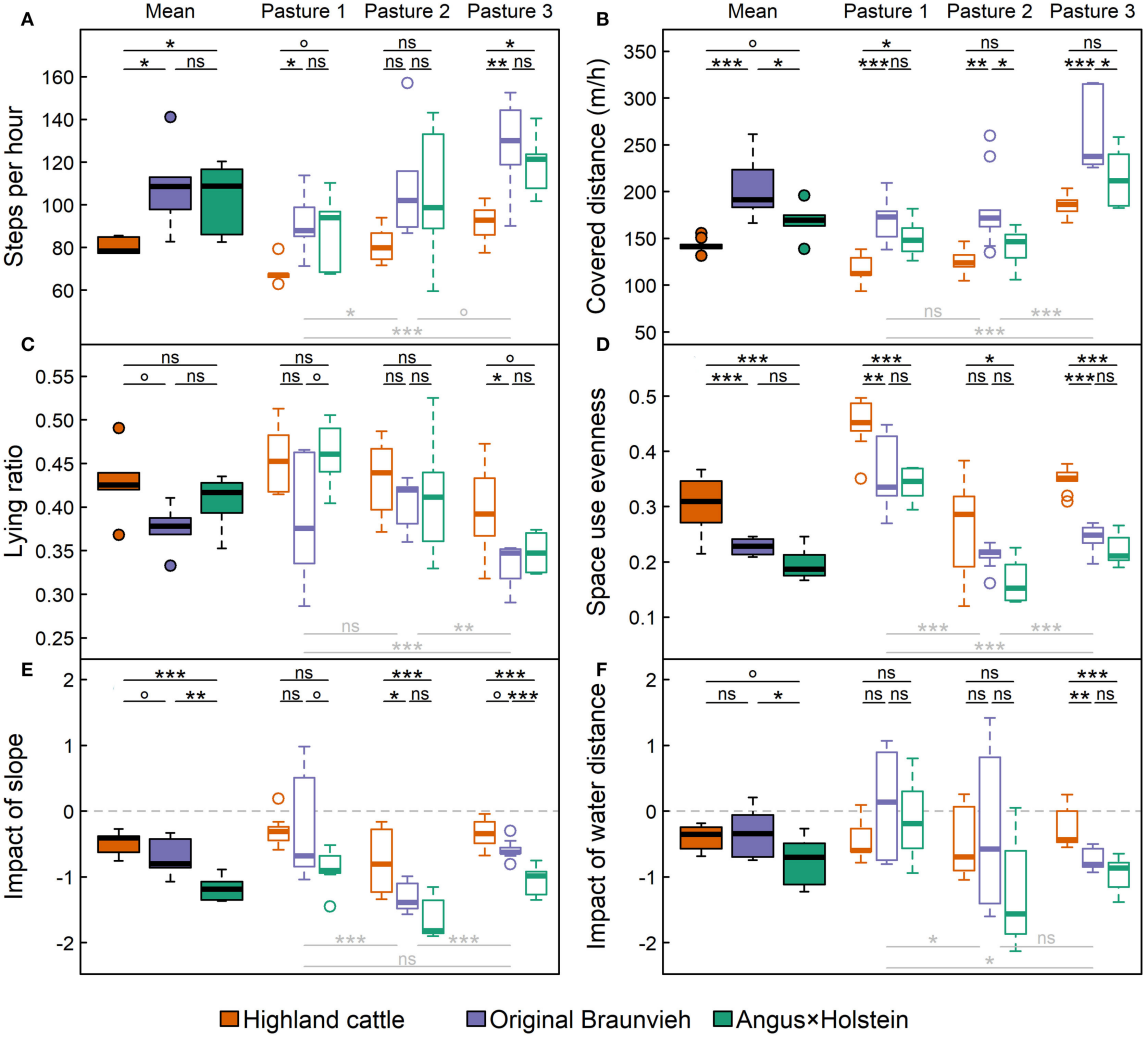
Movement behavior of the three breeds Angus × Holstein, Original Braunvieh, and Highland cattle: **(A)** The average number of steps recorded per hour; **(B)** the average covered distance per hour (i.e., the speed); **(C)** the ratio of the time spent lying; **(D)** the evenness of space use; **(E)** the impact of slope, and **(F)** of the distance to water points on cattle movement behavior. Steps and lying ratio were recorded for six, covered distance and space use evenness for all nine cows per breed. Filled boxplots represent mean values, empty boxplots differentiate by the three types of alpine pastures: (1) nutrient-rich, flat pasture, (2) heterogeneous dwarf-shrub pasture, (3) nutrient-poor fen and wood pasture. Significances of differences among breeds are displayed above the boxplots in black, those among pastures below the boxplots in light gray [box: 25th to 75th quartile range (IRQ); line: median; whiskers: max. 1.5 × IQR; points: outliers; ^ns^*p* > 0.1; °*p* < 0.1; *p < 0.05; **p < 0.01; ***p < 0.001].

The number of steps recorded by pedometer and the distance covered per hour showed similar patterns for the breeds and the pastures (Figures 5A,B): Original Braunvieh moved most (on average 4.6 km and 2,660 steps per day), followed by Angus × Holstein (4.1 km; 2,510 steps), which differed marginally from each other (steps: *p*_OB~*AH*_ = 0.86; distance: *p*_OB~*AH*_ = 0.02; displayed in black in Figure 5). Highland cattle (3.4 km; 1,880 steps) took significantly fewer steps than Angus × Holstein (*p*_HC~*AH*_ = 0.04) and Original Braunvieh (*p*_HC~*OB*_ = 0.02), covered less distance (*p*_HC~*AH*_ = 0.09; *p*_HC~*OB*_ < 0.001, respectively), and spent more time lying than the other two breeds (Figure 5C).

The linear regression model demonstrated that paddock size as well as the breed had a significant impact on the steps taken (*p*_breed_ < 0.001; *p*_size_ < 0.001) and the distance covered (*p*_breed_ < 0.001; *p*_size_ < 0.001), but interactions were not significant (steps: *p*_breed~*size*_ = 0.72; distance: *p*_breed~*size*_ = 0.10). All breeds were significantly less active on the small, nutrient-rich pasture 1 than on the large, nutrient-poor pasture 3 (steps: *p*_pasture_ < 0.001; distance: *p*_pasture_ < 0.001, displayed in gray in Figure 5) and spent more time lying there (*p*_pasture_ < 0.001). However, apart from this general trend, Highland cattle moved least on all pastures. For instance, on pasture 3 Highland cattle took about as many steps (Figure 5A) and covered about the same average daily distance (Figure 5B) as the other two breeds on pasture 1, where Angus × Holstein and Original Braunvieh moved least.

Furthermore, the evenness of space use differed among breeds (Figure 5D): Highland cattle used the pastures most evenly, whereas the space use of Angus × Holstein was more tightly clustered. The latter explored the available area least. There were no significant differences in evenness of space use between Angus × Holstein and Original Braunvieh (*p*_OB~*AH*_ = 0.2), but both breeds differed significantly from Highland cattle (*p*_HC~*AH*_ and *p*_HC~*OB*_ < 0.001). Similar to recorded steps and covered distance, the linear regression model demonstrated an impact of pasture size (*p*_size_ = 0.005) indicating that animals spread more evenly across smaller pastures. Thus, the relatively homogeneous, flat pasture 1 was used more evenly than the heterogeneous pastures 2 and 3 (both *p*_pasture_ < 0.001). However, when taking pasture size into account, the breed effect was more distinct (*p*_breed_ < 0.001) than the pasture size effect. The interaction of breed and size was insignificant (*p* = 1.0).

Steep slope generally reduced space use, but its impact differed among breeds (Figure 5E). Highland cattle avoided steep areas least, Angus × Holstein most clearly (*p*_HC~*AH*_ < 0.001). Original Braunvieh took an intermediate position (*p*_HC~*OB*_ = 0.07 and *p*_OB~*AH*_ = 0.001). On the flat pasture 1, the breeds differed only marginally in their response to slope. On pastures 2 and 3, which offered both, steep and flat areas, Highland cattle differed significantly from the other two breeds (pasture 2: *p*_HC~*OB*_ = 0.01, *p*_HC~*AH*_ < 0.001; pasture 3: *p*_HC~*OB*_ = 0.08, *p*_HC~*AH*_ < 0.001).

Moreover, the space use of cattle was influenced by the distance to water points (Figure 5E). The further a location was away from water, the less frequently it was visited. The impact of the distance to water increased with pasture size: on the small pasture 1, cattle were less influenced by the distance to water than on the largest pasture (*p*_pasture_ = 0.04). Clearer differences among breeds were observed in larger paddocks. On pasture 3, breeds differed significantly in their response to water distance. Highland cattle moved further away from water than Original Braunvieh (*p*_HC~*OB*_ = 0.002) and Angus × Holstein (*p*_HC~*AH*_ < 0.001).

### Differences in Foraging Behavior Among Breeds as Influenced by Pasture Conditions

We found differences in the evenness of forage selection and the forage quality of selected plant species among cattle breeds, indicating that different breeds preferred different groups of plants. For all averaged indicators, Highland cattle differed significantly from the other two breeds (*p* < 0.02). In contrast, no significant differences were found between Angus × Holstein and Original Braunvieh for any of the indicators of foraging behavior (*p*: 0.84–1).

**Figure 6:**
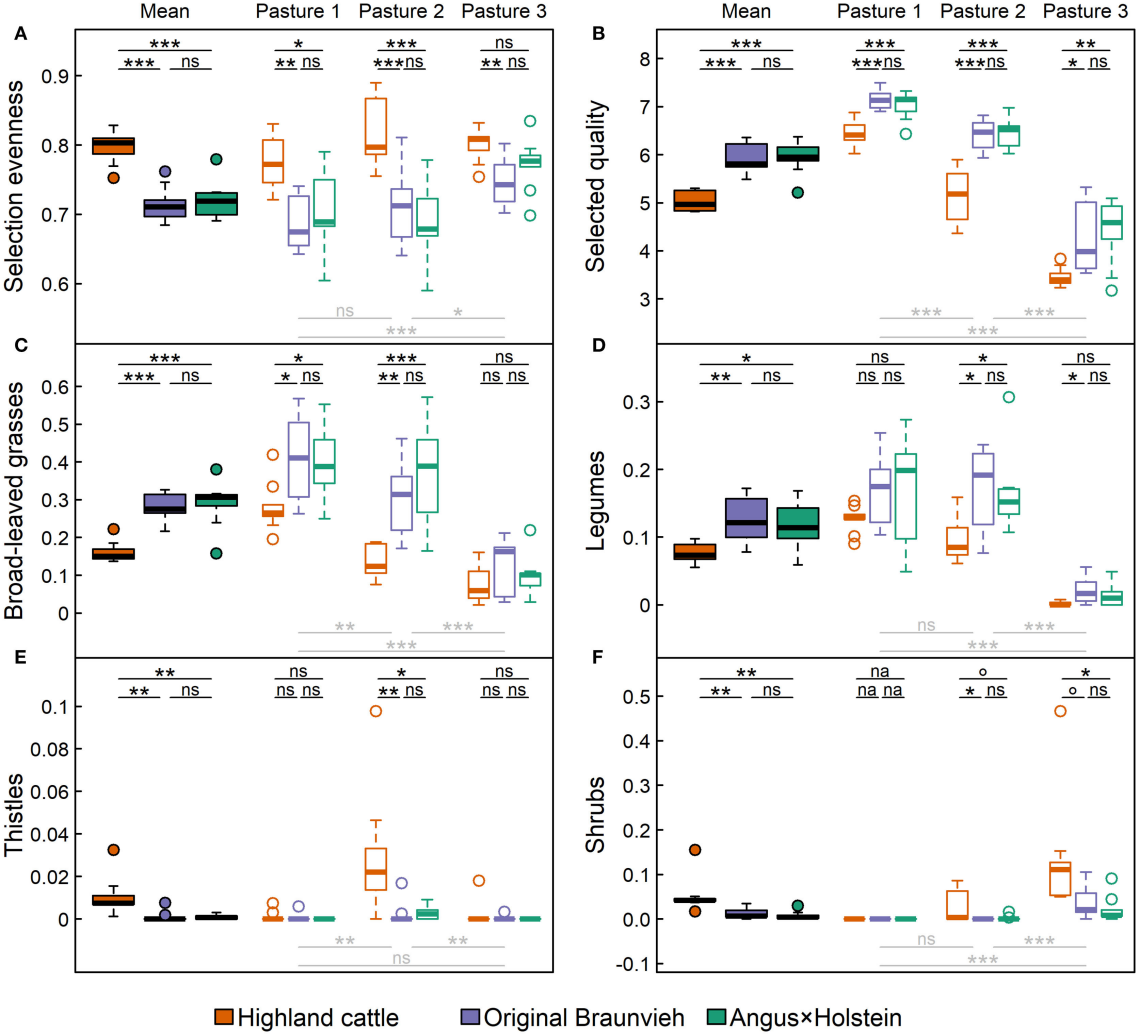
Forage selection behavior of three cattle breeds Angus × Holstein, Original Braunvieh, and Highland cattle. For all the nine cows per breed **(A)** the evenness of forage selection, **(B)** the average forage quality of the selected plants (21), and the share of **(C)** broad-leaved grasses, **(D)** legumes, **(E)** thistles, and **(F)** shrubs within the selected forage plants were measured. Filled boxplots represent average values, empty boxplots differentiate by the three types of alpine pastures: (1) nutrient-rich, flat pasture, (2) heterogeneous dwarf-shrub pasture, (3) nutrient-poor fen and wood pasture. Significances of differences among breeds are displayed above the boxplots in black, those among pastures below the boxplots in light gray [box: 25th to 75th quartile range (IRQ); line: median; whiskers: max. 1.5 × IQR; points: outliers; ^ns^*p* > 0.1; °*p* < 0.1; *p < 0.05; **p < 0.01; ***p < 0.001, na, not available].

Highland cattle foraged more evenly than the other breeds (Figure 6A), as observed in the overall average (*p* < 0.001), as well as in pasture-wise values. Only the evenness of forage selection by Highland cattle in pasture 3 did not differ significantly from Angus × Holstein cattle. Simply put, Highland cattle ate what was available. Thereby, they selected forage with significantly lower quality than the other two breeds (*p* < 0.001; Figure 6B). This was also reflected in breed-specific preference and avoidance of certain plant groups. Broad-leaved grasses and legumes were the plants with the highest forage quality in our study area. Angus × Holstein and Original Braunvieh had a stronger preference for these plants than Highland cattle (Figures 6C,D). In contrast, thistles and shrubs had the lowest forage quality and were foraged much less by Original Braunvieh and Angus × Holstein than by Highland cattle (Figures 6E,F). Since thistles primarily grew on pasture 2 and shrubs on pastures 2 and 3, differences were only detectable on these pastures.

In addition to breed, the pasture type also influenced foraging behavior: Cattle selected their forage more evenly on the homogeneous, nutrient-rich pasture 1 than on the heterogeneous, nutrient-poor pasture 3 (*p*_pasture_ = 0.001). In contrast, the quality of selected forage was highest on pasture 1, where plants with the highest forage quality grew, and lowest on pasture 3, where only forage of low quality was available (*p*_pasture_ < 0.001). Plant groups were grazed most on the pastures where they were most abundant: broad-leaved grasses and legumes were foraged significantly more often on pasture 1 than on pasture 3 (both *p*_pasture_ < 0.001), thistles were foraged more on pasture 2 than on pasture 1 (*p*_pasture_ = 0.003) and on pasture 3 (*p*_pasture_ = 0.004), and shrubs were consumed most on pasture 3 (*p*_pasture_ < 0.001).

### Allometry of Anatomy, Movement, and Foraging Behavior

**Figure 7:**
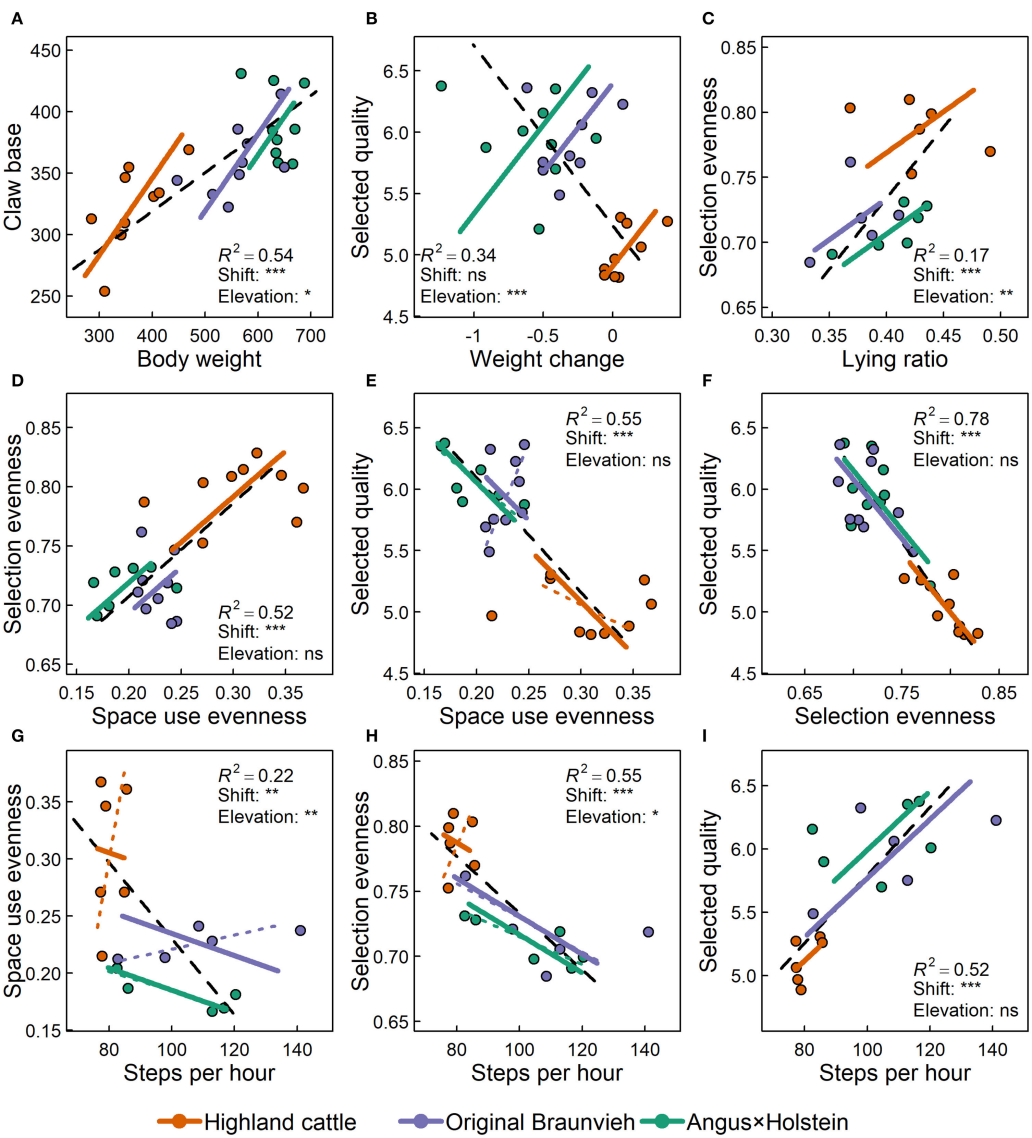
Selected allometric relationships among variables concerning anatomy, movement and foraging behavior of three cattle breeds. Panels **(A–I)** show allometric relationships of body weight (kg), claw base (cm), average daily body weight change over 10 weeks on alpine pastures (kg/d), the average number of steps recorded per hour, the ratio of the time spent lying, the evenness of space use, the evenness of plant species selection, and the average forage quality of the selected plants (21). The number of recorded steps and lying ratio were available for six cows per breed and the other variables for nine cows per breed. Figures show the overall allometric line for all animals (dashed black) with their regression coefficient (*R*^2^) as well as allometric lines for each of the three breeds. For all allometries where the slope differed significantly among breeds, the breed-specific allometric lines are provided (dashed lines) together with the forced common slope (solid lines). This was necessary for testing shift and elevation, for which significances of differences among breeds are given (^ns^*p* > 0.1; *p < 0.05; **p < 0.01; ***p < 0.001).

There were various strong allometric relationships among the variables tested (Figure 7), indicating that characteristics are consistently related to one another within each individual. However, most allometries were better explained, when breed was taken into account. As described above, Highland cattle differed from Original Braunvieh and Angus × Holstein in all variables measured, as indicated by a significant shift along the allometric lines (i.e., data clouds in Figure 7 are shifted along the direction of the lines). In addition to the simple positive or negative relationships, there were numerous effects of cattle breed on the specific allometries itself: We found significant differences in elevation among breeds' allometric lines (i.e., a parallel shift of the lines) for five out of nine allometries investigated.

Body weight and claw base (Figure 7A) were highly related to each other (*R*^2^ = 0.54). The relationship was similar for all three breeds, as indicated by the lack of significant differences in slopes of the breeds' allometric lines. Thus, heavy animals consistently had larger claws than light animals, independent of breed. However, breeds significantly differed in weight and claw base as indicated by a significant shift (*p* < 0.001) of Highland cattle data along the allometric lines compared to the other two breeds, which did not differ significantly from each other. In addition, not only the position of the point clouds of the breeds along the allometric lines, but also the elevation of their lines differed (*p* = 0.01). Highland cattle had significantly larger claw base in relationship to the body weight than the other two breeds.

There was an overall negative relationship between the average daily change in body weight and the quality of the selected forage (*R*^2^ = 0.34, Figure 7B): Animals that selected forage of higher quality lost more weight. Taking breeds into account reveals that this is primarily a breed effect, as indicated by the highly significant differences in elevation of the allometric lines (*p* < 0.001) and by the positive relationship within each breed, contrary to the overall negative relationship. In contrast to the other breeds, Highland cattle increased body weight despite low forage quality.

Breed also strongly affected the allometric relationship between selection evenness and lying ratio (*R*^2^ = 0.17, Figure 7C). In general, animals that selected their forage more evenly, spent more time lying. Forage selection was most even for Highland cattle and they spent the most time lying (shift: *p* < 0.001); however, but relative to the evenness of their forage selection, the lying ratio was low (elevation *p* = 0.005).

Space use evenness showed a positive relationship with selection evenness (*R*^2^ = 0.52, Figure 7D) and a negative relationship with the selected forage quality (*R*^2^ = 0.55, Figure 7E), which in turn was negatively linked to selection evenness (*R*^2^ = 0.78, Figure 7F). Animals that used space evenly also selected forage plants evenly, but they foraged plants of lower quality. Highland cattle used space and foraged most uniformly, but selected forage of lowest quality (shift of all allometries *p* < 0.001).

Over all animals, the average number of steps recorded per hour was negatively related with the evenness of space use (*R*^2^ = 0.22, Figure 7G). Animals that walked a lot covered less space. However, within each breed, the linkage of steps and space use evenness was less clear, pointing to a breed effect instead of a real allometric relationship (elevation: *p* = 0.001).

Finally, the number of steps recorded had a negative relationship with the evenness of selection (*R*^2^ = 0.55, Figure 7H) and a positive relationship with the quality selected (*R*^2^ = 0.52, Figure 7I). Animals that moved a lot, selected their forage plants more strictly and ingested forage of higher quality, irrespective of breed. Highland cattle, the breed that walked least, selected plant species least strictly, and of lowest quality (shift of both allometries *p* < 0.001). The significant differences in elevation (*p* = 0.02) among breeds' allometric lines show that Highland cattle would have foraged more selectively or taken fewer steps, if the relationship of steps and selectivity only depended on the individual.

## Discussion

As initially hypothesized, this comparative study of cattle on alpine pastures identified several close relationships among anatomy, movement, and foraging behavior, as demonstrated by allometric line fitting (hypothesis 1). Moreover, a considerable part of the variation among individuals is explained by breed, as indicated by Tukey range tests and by tests for allometric shift and elevation (hypothesis 2). Finally, the gradient of productivity from low-productive Highland cattle to intermediate Original Braunvieh to high-productive Angus × Holstein was consistently reflected in almost all parameters analyzed (hypothesis 3).

### Anatomical Differences Among Breeds and Consequences for Animal Health, Soil, and Vegetation

Body weight and claw base were closely related at the individual level: The heavier a cow was, the larger was the area of its claw base. However, breed did also matter: Relatively small claws were measured for the two high-productive breeds compared to those of Highland cattle. Therefore, the static pressure of body mass on every square centimeter of claw base was relatively high for Angus × Holstein, marginally less for Original Braunvieh, and significantly lower for Highland cattle. The similar weight-claw allometry of the two productive breeds goes along with Tuohy et al. (33), who found only small differences in weight-claw allometry between Holstein and Holstein × Jersey dairy cows. The relatively large claws of Highland cattle have been presumed (34), but have never been quantified in a comparative assessment. For this experiment, cows where kept under similar, but not identical housing condition over winter. To increase comparability, they grazed the same grounds over a period of 10 weeks prior to the claw measurement. An explanation for the differences observed among breeds may be that the breeding process increased cattle's body weights to a much larger extent than their claw bases—likely because nobody declared “large claws” as a breeding objective. These differences may strongly affect the animals as well as the pastures they graze.

On the one hand, huge body mass on a small base has the potential to affect claw health and may be an overlooked source of claw pathologies. Previous studies did not find differences in claw health among high-productive breeds (26, 35). However, testing a broader range of productivity, low-productive dairy breeds showed significantly fewer claw diseases than high-productive breeds (36). This may, at least partially, be explained by differences in allometry between body weight and claw base, since less weight burdens each square centimeter of claw. Correspondingly, many Highland cattle farmers reported that they almost never observe claw diseases and rarely need claw trimming or veterinary assistance at their home farms. Unfortunately, the relative frequency of claw diseases in Highland cattle has never been analyzed relative to other breeds.

On the other hand, claw pressure not only has an impact on animal welfare, but also on pastureland. Generally, heavy animals on relatively small claws compress the soil more forcefully, thereby promoting erosion (37). Herbin et al. (38) reported an increase in soil penetration resistance and a decrease in porosity on pastures grazed by heavy animals with relatively small claw base. Accordingly, we found more open ground susceptible to erosion in pastures of high-productive breeds than in those of Highland cattle in a previous study (13). If grazing intensity increases, to which trampling pressure contributes, soil organic carbon decreases, with negative consequences for greenhouse gas emissions (39). High trampling pressure comes along with structural deterioration and compaction of soil (40), whereas water storage capacity and pasture productivity decrease (41). The negative effects of trampling (42) are particularly notable where heavy animals are present on steep slopes (43). In contrast, light Highland cattle with large claws have the potential to minimize trampling-induced erosion effects, especially on shallow alpine soils that benefit notably from light and moderate grazing (44).

Moreover, trampling pressure is a driver of selection and thus affects vegetation composition by promoting plant species well-adapted to trampling (45, 46): (i) Short plants with caespitose, matted or rosette architecture and with elastic tissue are less damaged; (ii) prostrate or stoloniferous species with rooting stems or stolons can regrow from intact parts after trampling; (iii) species with high regenerative capacity can quickly rebuild damaged parts; (iv) early bloomers avoid being trampled by finishing their life cycle before the first grazing in spring. These plant species become dominant under high trampling impact (47). As a result, they are significantly more frequent on pastures of high-productive breeds than on Highland cattle pastures (13). On pastures of heavy animals with relatively small claws, trampling pressure is an important ecological driver of vegetation composition and trampling-adapted plants outcompete less adapted species, resulting in a decrease in plant species richness (13).

### Movement Behavioral Characteristics Are Allometrically Related at the Breed Level

Soil and vegetation is not only affected by static pressure, but also by the frequency of trampling and its spatial distribution: The static pressure, as measured in the present study, only applies when the animal is standing, equally weighting all four feet. Since pressure concentrates onto three or even two claws while moving, trampling pressure increases as the cow walks and exerts additional destructive kinetic energy (42). As measured by pedometer and GPS tracking in our study, Highland cattle moved least and slowest (i.e., they covered least distance per time) on almost all pastures. Pressure on vegetation and soil is less intensive and less frequent and thereby, the negative impact of trampling on soil and vegetation described above may be reduced on Highland cattle pastures.

Generally, cattle do not cover available space evenly, especially on heterogeneous alpine pastures (10, 23, 48). It seems logical that animals that walk less visit fewer parts of the pasture and leave most places undiscovered. Yet, the opposite was the case: The fewer steps an animal took, the more evenly it occupied the available space. This unexpected negative allometry makes sense, if the breed effect is considered. Despite their slowness, Highland cattle visited the most distant and steepest places on the pastures. In contrast, Original Braunvieh and Angus × Holstein took many steps, but explored a smaller share of the available area. The sparse flat and nutrient-rich parts of the pastures, where they spent most time, provide plants of high forage quality and smooth terrain, which are both attractive qualities (23, 48), especially for cattle with high nutritive demand and large body size. The data suggest that both productive breeds moved more than Highland cattle, but within a smaller space, in flatter areas and closer to water points. Undemanding Highland cattle gathered less frequently on the attractive, flat parts of the pastures, although pasture size was large enough not to force them to forage on the poorer, steep parts of the pastures far away from water. A more even space use is expected in smaller paddocks and at higher stocking density (49), but Highland cattle spread more evenly than would be expected based on paddock size and stocking. The differences in movement behavior among breeds go along with Spiegal et al. (50), who found a traditional cattle breed visiting more different places than a high-productive breed, which preferred the hotspots more clearly. As Highland cattle spread more evenly, they comply with farmers' ambitions to utilize remote or unattractive parts of their land.

Although paddocks of Highland cattle were about one third smaller, it is unlikely that differences in movement behavior among breeds were caused by paddock size alone: Highland cattle in paddocks of pasture 2 moved less than Angus × Holstein and Original Braunvieh in the smaller paddocks of pasture 1. Moreover, in pasture 3, Highland cattle moved about as much (93 steps and 190 m per hour), and as evenly (Camargo's index of evenness: 0.35) as the other breeds did on pasture 1 (steps: 95 and 91; distance: 150 and 170 m per hour; Camargo's index: 0.35 and 0.34 for Angus × Holstein and Original Braunvieh, respectively), although Highland cattle had nearly three times more space in pasture 3 than the other breeds in pasture 1. If movement were inhibited by paddock size, Highland cattle would have taken more steps, covered more distance and spread less even across pasture 3. Additionally, if the movement were a function of paddock size alone, the breed differences should be expected to diminish with increasing available area. However, the opposite was observed: The breed effect on movement parameters was stronger in the large paddocks of pastures 3 than in the small paddocks of pasture 1. In pasture 3, where the differences were most significant, the three cows of each breeds had access to more than 1 ha pastureland and were thus hardly limited in their movement. Nevertheless, Highland cattle covered the least distance and spread most evenly there. Finally, the linear regression model clearly demonstrated a breed effect that goes beyond the effect of pasture size.

### Foraging Behavior Depends on Breeds' Level of Productivity

Generally, animals that used space evenly also foraged evenly, as supported by Bailey et al. (51), and cattle that walked little also selected forage plants evenly. Independent of the breed, an individual cow that spread evenly, grazed many different plants, and took only few steps. This suggests that a highly selective cow needs to cover more distance to find the most palatable plants, while a less selective cow eats what is in close proximity of her mouth, not caring much about the quality. This assumption corresponds with the low quality of the selected forage for those animals that took only few steps. Highland cattle moved the least, thereby foraging most evenly and selecting a diet of lowest quality compared to the other two breeds. Original Braunvieh cattle took an intermediate position, but were much more similar to Angus × Holstein than to Highland cattle. Differences in the quality of the selected forage may be additionally explained by cattle's physical access to steep slopes (10), which typically offer forage of lower quality. While large body mass may hinder high-productive breeds' ability to visit steep areas, Highland cattle can reach them and forage the poorer forage there.

Through modern breeding, Original Braunvieh and Angus × Holstein have been selected for a higher growth rate and milk production than Highland cattle (15). Therefore, they are in need of high-nutritive forage, such as broad-leaved grasses and legumes (21) and move longer distances to reach these plants. In contrast, the lower nutritive demand of slow-growing Highland cattle were covered by forage of lower quality. Thus, they save steps while foraging.

In the long term, the higher selectivity of more productive breeds has important consequences for pasture vegetation (13). Unattractive plants co-evolved under grazing pressure and developed strategies to avoid foraging. Thus, toxic species (e.g., *Ranunculus, Aconitum*), plants of low forage quality (e.g., *Nardus stricta*), plants with physical defense mechanisms (e.g., thistles, *Deschampsia cespitosa*), or shrubs are avoided by cattle. The more selectively herbivores graze the more dominant these species become (52, 53). Since they outcompete other plants less-adapted to grazing, plant species richness decreases. Accordingly, significantly fewer plant species were found on pastures grazed by high-selective, high-productive breeds than on pastures of less selective Highland cattle (13).

Interestingly, cattle that foraged more evenly spent more time lying. A diet that is chosen evenly across the pasture contains more fiber-rich plants with higher leaf dry matter content and smaller specific leaf area than a strongly selected diet (14). Fiber increases the ruminal retention time and, hence, the time required to digest the forage (54). Therefore, an animal that forages evenly, selects a diet of lower digestibility and, subsequently, spends longer time ruminating, normally done while lying. Highland cattle that foraged most evenly and selected plants of lowest digestibility, spent the longest time lying due to increased ruminal retention time. In addition to the overall allometric relationship of selection evenness and lying time concerning all individuals, there was a clear breed effect as indicated by the difference in elevation: If the relationship were independent of breed, Highland cattle would have lain even more, indicating that Highland cattle digested relatively quickly with respect to the quality of their forage. This suggests that Highland cattle have a more effective food conversion than higher-productive breeds. A more efficient food conversion of less productive breeds was shown in previous, comparative experiments for beef breeds (18, 55, 56) and dairy cattle (57, 58). Morris and Wilton (59) showed that small beef and dairy cattle are more efficient in weight gain and milk production, respectively, than large cattle. Accordingly, Highland cattle seem to make use of fiber-rich and nutrient-poor forage more efficiently and may, therefore, be better adapted to the harsh environment of alpine pastures than high-productive breeds. As a result, Highland cattle were able to gain body weight, even on the nutrient-poor pastures of our study area, where both of the production-oriented breeds lost weight.

Additionally, the low average temperature of 10.0°C (SD: 5.0°C, range: −2.8 to 21.8°C) in the study area during the study time forced cattle to invest thermal energy. Highland cattle may save energy because of their woollier fur, which provides better insulation than the short fur of Original Braunvieh or Angus × Holstein cattle.

Finally, the positive weight gain of Highland cattle may be promoted by more efficient movement and foraging behavior. By selecting plant species more evenly and consequently moving about one quarter less and lying more, Highland cattle save legwork and kinetic energy. Moreover, they have to move significantly less body mass with each step. Thereby, Highland cattle balance the lower nutrient content of their diet.

Technically, the positive weight gain of Highland cattle could result from a higher dry matter intake. This parameter was not measured, but visual observation indicated rather smaller than larger bites and bite rates for Highland cattle. This goes along with Fraser et al. (56), who found a higher weight gain despite smaller dry matter intake for a traditional breed in comparison to a high-productive beef breed.

The findings of this study suggest that anatomical characteristics as well as movement and foraging behavior depend on the level of breeding intensity. The differences among breeds arose during the breeding process, since the underlying mechanisms of artificial selection do not differ from natural selection: Populations adapt to drivers of selection. The more important a criterion is for the reproductive success, the more clearly the population will evolve with respect to this trait (60). By strictly selecting for milk or meat yield, breeders establish strong selective forces that unintentionally override many traits less focused on. Characteristics that are less important for reproductive success (i.e., breeders do not select for them) are subordinated to stronger drivers. Subsequently, if there is no evolutionary pressure for a certain trait, it will alter or disappear unintentionally (61). If, for example, breeders do not select for efficient conversion of fiber-rich fodder, efficiency becomes a less essential driver of reproductive success and subsequently decreases. Instead, cattle adapt to nutrient-rich and concentrated feed.

### Implications for Management, Breeding, and Biodiversity

The general tendency of cattle to avoid plant species of low forage quality (14) and the places where such plants are dominant (23) counteracts pasture improvement and maintenance. To reduce the abundance of weeds and shrubs and thereby maximize pasture use, cattle should ideally forage all plants and evenly visit all parts of a paddock. Usually, alpine grasslands are so heterogeneous that cattle almost inevitably use it unevenly (23). Highland cattle, which grazed most evenly among the breeds investigated, were able to exploit even unattractive plants and places.

The breed differences in space use evenness, in impact of slope, and in impact of the distance to water were most evident on pasture 3, which was more heterogeneous and offered poorer forage quality than the two other pastures in the experiment. This observation emphasizes the benefit of undemanding breeds, especially for grasslands that are unsuited for modern agricultural management (62).

It is indisputable that the production output of Highland cattle is low. Under intensive housing conditions, they cannot compete with the growth rate and carcass weight of other breeds (15). Their real advantage is to cope with unfavorable conditions. This is highlighted by the small, yet existent increase in body weight of Highland cattle during the experiment, whereas the other breeds lost weight due to the poor nutritive supply. Though modern breeds have a higher weight gain potential, they cannot reach it on nutrient-poor pastures. Therefore, grazing such areas with high-productive breeds is economically inefficient due to the loss of body weight. In contrast, Highland cattle, which grow less effective and efficient in intensive farming systems, are still able to create a small output under poor conditions, resulting in a positive cost-value ratio (63).

In this experiment, low-productive breeds were represented by Highland cattle, but there are many other low-demanding and low-productive breeds in most European mountain regions. Among these are Tarentaise, Valdostana Castana, Vosgienne, Hinterwaelder, Grauvieh, Murbodner, Galloway, Dexter, and numerous others. Like Highland cattle, these local breeds are adapted to grazing nutrient-poor pastures and thereby, contribute to sustaining semi-natural grasslands unsuitable for high-productive breeds. Using local livestock also provides cultural ecosystem services by maintaining cultural heritage and genetic diversity of livestock. Although it remains to be tested whether other low-productive cattle breeds behave similarly to Highland cattle, this study demonstrates a strong effect of breeds' productivity on numerous traits neglected by output-oriented breeding. This suggests that other low-productive breeds may also be appropriate for grassland conservation.

Breeders of low-productive cattle are proud of the benefits their animals provide, including high robustness, soil protection, reduction in problematic plant species, increased biodiversity, and a general efficiency even in these low-productive systems. Breeders should bear in mind that these qualities are closely related to the low productivity of this breed. Although it is tempting to modify breeding aims toward higher output, our data suggest that if Highland cattle were bred more productively, many of these benefits would be lost, as has been the case with other breeds. On the other hand, breeders of high-productive cattle may consider differences among individuals as a potential to increase production efficiency (9, 10).

In mountainous regions, pasture biodiversity is not only under general pressure of climatic and socio-economic changes (64–66). The structural changes in modern agriculture have also negatively affected low-productive grasslands: In conjunction with poor forage quality, pastures and meadows that are difficult to manage due to steep slope, too-wet or too-dry conditions become unattractive to farmers of high-productive cattle, because these animals cannot exploit their genetic potential under these conditions, as demonstrated by Bovolenta et al. (67). Therefore, the intensity of management decreases, and pastures are eventually abandoned (68, 69). As a consequence, the rich biodiversity of European mountainous pastures suffers, for example, from the continuous spread of shrubs and wood on formerly diverse and open grasslands (17, 70–72). Although biodiversity conservation has begun to receive increasing attention as an important ecosystem service of alpine pastures (73), not even public financial support for mountain farmers is currently able to halt the abandonment of low-productive pastures (74). An appropriate use of these habitats is grazing with undemanding livestock, such as goats, sheep, or low-productive cattle breeds. There is no need for farmers to change their entire livestock, but some low-productive animals can often be added to existing herds without difficulty, as they are undemanding, not only in forage quality, but also in housing conditions. Incorporation of low-productive cattle breeds is, therefore, a key strategy to use low-productive grasslands efficiently and to conserve their biodiversity.

## Data Availability Statement

The datasets presented in this study can be found in the online repository Zenodo https://zenodo.org/record/3707638.

## Ethics Statement

This animal study was reviewed and approved by Veterinary Office of Grisons (authorization GR2018_12). Written informed consent was obtained from the owners for the participation of their animals in this study.

## Author Contributions

CP, JI, JB, TB, and MS conceived the ideas and designed the methodology. CP and MS collected the data, analyzed the data, and led the writing of the manuscript. All authors contributed critically to the drafts and gave final approval for publication.

## Conflict of Interest

The authors declare that the research was conducted in the absence of any commercial or financial relationships that could be construed as a potential conflict of interest.
